# A phase 1, first-in-child, multicenter study to evaluate the safety and efficacy of the oncolytic herpes virus talimogene laherparepvec in pediatric patients with advanced solid tumors

**DOI:** 10.3389/fped.2023.1183295

**Published:** 2023-05-24

**Authors:** Lucas Moreno, Pierre Teira, James M. Croop, Nicolas U. Gerber, Nicolas André, Isabelle Aerts, Luis Gros Subias, Bram De Wilde, Francisco Bautista, Brian Turpin, Srinivasa Kunduri, Ali Hamidi, Tatiana Lawrence, Keri A. Streby

**Affiliations:** ^1^ Hospital Universitari Vall d’Hebron, Barcelona, Spain; ^2^ Centre Hospitalier Universitaire Sainte-Justine, Montreal, Canada; ^3^ Division of Hematology and Oncology, Riley Hospital for Children, Indianapolis, IN, United States; ^4^ Department of Oncology, University Children’s Hospital, Zurich, Switzerland; ^5^ SMARTC Unit Centre de Recherche en Cancérologie de Marseille, Inserm U1068, Aix Marseille University, Marseille, France; ^6^ Service d'Hématologie & Oncologie Pédiatrique, Timone Hospital, AP-HM, Marseille, France; ^7^ Institut Curie, Paris, France; ^8^ Universitair Ziekenhuis Gent, Ghent, Belgium; ^9^ Division of Pediatric Hematology and Oncology, Hospital Universitario Niño Jesús, Madrid, Spain; ^10^ Cincinnati Children’s Hospital, Cincinnati, OH, United States; ^11^ Parexel, Hyderabad, Telangana, India; ^12^ Amgen Inc., Thousand Oaks, CA, United States; ^13^ Department of Hematology/Oncology/BMT, Nationwide Children's Hospital/The Ohio State University, Columbus, OH, United States

**Keywords:** immunotherapy, pediatric solid tumor, talimogene laherparepvec, non-CNS tumors, relapsed and refractory cancer, oncolytic herpes virus

## Abstract

**Background:**

The survival rates for pediatric patients with relapsed and refractory tumors are poor. Successful treatment strategies are currently lacking and there remains an unmet need for novel therapies for these patients. We report here the results of a phase 1 study of talimogene laherparepvec (T-VEC) and explore the safety of this oncolytic immunotherapy for the treatment of pediatric patients with advanced non–central nervous system tumors.

**Methods:**

T-VEC was delivered by intralesional injection at 10^6^ plaque-forming units (PFU)/ml on the first day, followed by 10^8^ PFU/ml on the first day of week 4 and every 2 weeks thereafter. The primary objective was to evaluate the safety and tolerability as assessed by the incidence of dose-limiting toxicities (DLTs). Secondary objectives included efficacy indicated by response and survival per modified immune-related response criteria simulating the Response Evaluation Criteria in Solid Tumors (irRC-RECIST).

**Results:**

Fifteen patients were enrolled into two cohorts based on age: cohort A1 (*n* = 13) 12 to ≤21 years old (soft-tissue sarcoma, *n* = 7; bone sarcoma, *n* = 3; neuroblastoma, *n* = 1; nasopharyngeal carcinoma, *n* = 1; and melanoma, *n* = 1) and cohort B1 (*n* = 2) 2 to <12 years old (melanoma, *n* = 2). Overall, patients received treatment for a median (range) of 5.1 (0.1, 39.4) weeks. No DLTs were observed during the evaluation period. All patients experienced at least one treatment-emergent adverse event (TEAE), and 53.3% of patients reported grade ≥3 TEAEs. Overall, 86.7% of patients reported treatment-related TEAEs. No complete or partial responses were observed, and three patients (20%) overall exhibited stable disease as the best response.

**Conclusions:**

T-VEC was tolerable as assessed by the observation of no DLTs. The safety data were consistent with the patients' underlying cancer and the known safety profile of T-VEC from studies in the adult population. No objective responses were observed.

**Trial Registration:**

ClinicalTrials.gov: NCT02756845. https://clinicaltrials.gov/ct2/show/NCT02756845.

## Introduction

Childhood cancer represents 5% of all childhood deaths and 15% of all deaths amongst those aged 5 to 14 years (1). Despite improvements in overall 5-year survival rates, the prognosis of relapsed and refractory pediatric solid tumors is poor and there remains an unmet need for novel therapies for such patients (2).

Compared with those in adult patients, solid tumors in pediatric patients exhibit biological and molecular differences, including fewer somatic mutations and fewer infiltrating T cells (3, 4). Recent studies have evaluated immune checkpoint inhibitors in the pediatric population (nivolumab [ADVL1412; NCT02304458], pembrolizumab [KEYNOTE-051; NCT02332668], atezolizumab [iMATRIX; NCT02541604], and avelumab [NCT03451825]), in which only 3% of patients with solid tumors experienced an objective response (5). The reason for the lack of efficacy of checkpoint inhibitor clinical trials in pediatric patients is speculated to be related to fundamental differences in the immunobiology of cancers in children compared with adults. Childhood cancers are generally considered “cold tumors,” with low mutational burdens and fewer infiltrating T cells. The continued study of the impact of immunotherapy on pediatric immunobiology is warranted (5, 6).

Therapies that recruit immune cells into the tumor microenvironment are attractive therapeutics for immunologically inert pediatric solid tumors (5). Oncolytic viruses are a class of immunotherapy that target cancer cells, cause an antitumor immune response, and are associated with a manageable safety profile (7). Some clinical trials have assessed the benefit of oncolytic viruses in a pediatric setting. Recent phase 1 trials with the Seneca Valley virus (NTX-010), reovirus (Reolysin), vaccinia virus JX-594 (Pexa-Vec), herpes simplex virus type-1 (HSV-1; Seprehvir), and Icovir-5 (Celyvir) have shown that these therapies are well-tolerated in pediatric patients with non–central nervous system (CNS) tumors; however, no objective responses were observed (8–12).

Talimogene laherparepvec (T-VEC) is a genetically modified HSV-1–based oncolytic immunotherapy that is administered by intralesional injection and is designed to target and kill cancer cells by direct lysis and stimulation of an adaptive antitumor immune response (13). The neurovirulence factor ICP34.5 and the ICP47-encoding genes are functionally deleted in the virus, while the gene for granulocyte macrophage colony-stimulating factor (GM-CSF) is inserted. The ICP34.5 functional deletion allows the virus to replicate selectively in tumors. The role of ICP47 is to block antigen presentation to major histocompatibility complex class I by blocking the transporter associated with antigen processing (TAP). This deletion also allows the increased expression of the *US11* gene. This promotes the growth of the virus in cancer cells without decreasing tumor selectivity (13).

The efficacy of T-VEC has been demonstrated in the pivotal phase 3, randomized, open-label OPTiM trial of T-VEC in adult patients with advanced melanoma, which showed significant improvement of the durable response rate over subcutaneous GM-CSF (16.3% vs. 2.1%; *P* < 0.001) (14). The most frequently experienced adverse events in the adult population were fatigue, chills, and pyrexia.

Outside melanoma, T-VEC was tested in mouse xenograft models of Ewing sarcoma (A-673), neuroblastoma (SK-N-AS), osteosarcoma (SJSA-1), rhabdoid tumor (G-401), and rhabdomyosarcoma (SJCRH30). Across these models, 65% to 100% of tumor growth inhibition was observed and 10% to 30% of the animals showed complete tumor regression (15). These data are consistent with reports in the literature of the efficacy of other HSV-1 oncolytic viruses and support the evaluation of T-VEC in pediatric patients (16). We report the results from the primary analysis of the phase 1, multicenter, open-label, dose de-escalation study to determine the safety and tolerability of T-VEC administration in pediatric patients with advanced non-CNS tumors at the recommended adult dose.

## Patients and methods

### Patient characteristics

This study enrolled patients (2 to  ≤21 years of age) with histologically or cytologically confirmed non-CNS solid tumors that recurred after standard/frontline therapy (or for which no therapeutic options exist). Patients were enrolled into two cohorts based on age. Patients aged 12 to ≤21 years of age were eligible for cohort A1 and those aged 2 to <12 years were eligible for cohort B1. Tumors had to be suitable for intralesional injection. Visceral or bone lesions were not considered suitable for injection, with the exception of bone lesions that had a soft tissue component amenable to injection. Initially, patients had to have local HSV-1 serostatus available; however, this requirement to enroll patients according to HSV-1 serostatus was removed due to a low prevalence of HSV-1 seropositivity in the overall pediatric population (17). Patients must have had measurable or non-measurable disease. Patients in cohorts A1 must have had a Karnofsky play performance score of ≥70% and those in B1 were required to have a Lansky play performance score of ≥70%.

Patients must have had a life expectancy of greater than 4 months from the date of enrollment, adequate organ function, and proven to not be pregnant. Patients with a diagnosis of a hematological malignancy, primary ocular or mucosal melanoma, or history of other malignancy within the past 5 years were excluded. Also excluded were patients with active infections, those with a history or evidence of active immunosuppression, and those who had previously been treated with T-VEC or any other oncolytic virus.

### Study design

T-VEC was administered by an intralesional injection into cutaneous, subcutaneous, nodal, and other non-visceral tumors to evaluate its safety and efficacy in pediatric patients (Supplementary Figures S1–2). T-VEC was initially injected at 10^6^ plaque-forming units (PFU)/ml (≤4.0 ml total volume), followed by 10^8^ PFU/ml (≤4.0 ml total volume) on the first day of week 4. Subsequent injections of T-VEC were administered at 10^8^ PFU/ml (≤4.0 ml total volume) every 2 weeks. The study comprised a screening period of ≤8 days, a treatment period, a safety follow-up period up to 37 days after the last dose of the study treatment, and a long-term follow-up period during which patients were followed up to assess survival and the use of subsequent anticancer therapies every 12 weeks (±28 days) until death, consent withdrawal, or up to 24 months from the time the last patient enrolled, whichever occurred first. The study included a dose-limiting toxicity (DLT) evaluation period, which was 35 days from the initial dose of T-VEC, and a dose de-escalation phase using a standard “3 + 3” design (18).

T-VEC treatment continued until complete response (CR), disappearance of all injectable lesions, confirmed progressive disease (PD) per modified immune-related response criteria simulating Response Evaluation Criteria in Solid Tumors version 1.1 (irRC-RECIST 1.1) (19), intolerance of study treatment, need for alternative anticancer therapy, or 24 months from the first dose of T-VEC, whichever occurred first. Dose reduction within a cohort for an individual patient who experienced adverse events was not allowed. If a patient could not tolerate the full dose during the administration of T-VEC due to an injection-related adverse event, the total volume given was recorded and the reason for intolerance was documented as an adverse event. If treatment-related toxicities occurred (including ≥grade 2 immune-mediated adverse events, ≥grade 2 allergic reactions, or any other ≥grade 3 hematologic/non-hematologic toxicity), treatment was delayed until the toxicity was resolved to ≤grade 1 Common Terminology Criteria for Adverse Events (CTCAE) version 4.0 or baseline level. Treatment was withheld if the patient required corticosteroid dosing greater than that required for maintenance physiologic replacement therapy or demonstrated evidence of new or active CNS metastases. Reasons for permanent discontinuation of T-VEC included confirmed disease progression, a DLT during the DLT evaluation period, or a dose delay for more than 6 weeks as a result of adverse events. Blood, swabs, and urine samples were tested for T-VEC DNA by quantitative polymerase chain reaction (qPCR) analysis predose and/or postdose at cycles 1–4 and at safety follow-up.

The trial was registered as NCT02756845 and EudraCT 2015-003645-25. The study was conducted in accordance with the principles of the International Council for Harmonisation Good Clinical Practice guidelines and the World Health Organization's Declaration of Helsinki. The study protocol and subsequent amendments were reviewed and approved by the institutional review boards/ethics committees at each participating site (see Supplementary Table S1 for individual site names and addresses). All patients or parent/legal guardians provided written informed consent. The data cutoff for this primary analysis was January 17, 2022. T-VEC injection procedures and precautions followed the local approved label guidance (20). Secondary transmission of T-VEC has not been reported with proper administration and handling process (21, 22).

### Endpoints

The primary endpoint was to determine the safety and tolerability of T-VEC, as assessed by the incidence of DLTs. Secondary endpoints included overall response rate (ORR), duration of response (DOR), time to response (TTR), time to progression (TTP), progression-free survival (PFS) using modified irRC-RECIST version 1.1, and overall survival (OS). The additional safety endpoints were to evaluate the safety and tolerability of T-VEC through assessment of patient incidence of treatment-emergent and treatment-related adverse events. Treatment-emergent adverse events (TEAEs) were defined as any adverse event occurring after the first dose through 30 days after the last dose of T-VEC. Treatment-related adverse events were defined as TEAEs that were considered to be related to the treatment of T-VEC by the investigator. Planned exploratory endpoints included the patient incidence of clearance of T-VEC DNA from blood and urine, rate of detection of T-VEC DNA and virus (exterior of occlusive dressing, the surface of injected lesions, the oral mucosa, and in lesions suspected to be herpetic in origin), and antitumor activity of T-VEC in injected and uninjected lesions.

### DLT evaluation

DLTs that occurred within the DLT period of 35 days from the initial administration of T-VEC were defined as any grade 4 non-hematologic toxicity, grade 3 non-hematologic toxicity lasting for more than 3 days, grade ≥3 non-hematologic laboratory value requiring medical intervention/hospitalization or if the abnormality persisted for more than 1 week, grade 3/4 febrile neutropenia, thrombocytopenia, serious herpetic event, or grade 5 toxicity (i.e., death), or any intolerable toxicity requiring permanent discontinuation of T-VEC. Potential or known unintended exposure to T-VEC by healthcare providers and patients' close contacts and related suspected signs or symptoms of T-VEC exposure were recorded.

### Assessments

Radiographic imaging was done at baseline and at weeks 8 and 16. From then onwards, for patients remaining on treatment, imaging continued every 12 weeks until confirmed PD per modified irRC-RECIST or start of a new anticancer treatment. Radiographic imaging concluded at the safety follow-up visit.

Tumor response was assessed per modified irRC-RECIST and required response/PD was confirmed by a second consecutive clinical and radiographic assessment ≥4 weeks after the first documented response/PD.

Safety was assessed by patient incidence of adverse events and laboratory abnormalities (including potential hepatoxicity). The Medical Dictionary for Regulatory Activities version 24.1 was used to code adverse events to a system organ class (SOC) and a preferred term within the SOC. The severity of adverse events was graded using CTCAE version 4.0.

### Study oversight

This study was sponsored by Amgen Inc. All procedures received institutional approval. The trial was designed by employees of the sponsor in collaboration with the primary investigators. Data were collected by investigators and analyzed by statisticians employed by the sponsor. The dose-level review team (DLRT) reviewed safety and efficacy data once a year (2018, 2019, and 2021) to ensure no avoidable increased risk of harm to patients.

### Statistical analysis

With both safety and efficacy data of 13 patients available, the DLRT received endorsement from the European Medicines Agency to decrease the sample size from 18 to 24 to at least 13 patients due to the absence of clinical response and enrollment difficulties. The study closed with 15 patients enrolled, two were not DLT evaluable due to a treatment period of <35 days.

The primary analysis was conducted 35 days after the last patient was enrolled and received at least one dose of T-VEC (January 2022). For cohort opening and dose de-escalation, criteria from “3 + 3” phase 1 design assuming a true DLT incidence rate <33% was used. A dose level was considered safe for a cohort if <33% of all DLT-evaluable patients experienced a DLT (a minimum of six DLT-evaluable patients). The probability of declaring a dose level safe (unsafe) for a range of true DLT rates based on six DLT-evaluable patients was 89% (11%), 42% (58%), and 11% (89%) if the true DLT rate was 10%, 30%, and 50%, respectively.

Patient incidence of DLTs was summarized using the DLT analysis set, which included DLT-evaluable patients, defined as those who were followed-up for ≥35 days on treatment from the initial dosing and received ≥2 doses of T-VEC (except patients who had a DLT after the first dose) during the DLT evaluation period. The safety analysis set, defined as patients who received ≥1 dose of T-VEC, was used for the safety and efficacy analyses. ORR, DOR, TTR, TTP, PFS, and OS were summarized by cohort and in the overall population. ORR was summarized with an associated exact 95% confidence interval (CI). The DOR among responders, TTP, PFS, and OS were estimated using the Kaplan-Meier method. Summaries were provided for all safety endpoints as well as exposure to study treatment, and summary statistics were estimated for TTR among responders.

## Results

### Patients

**Figure 1:**
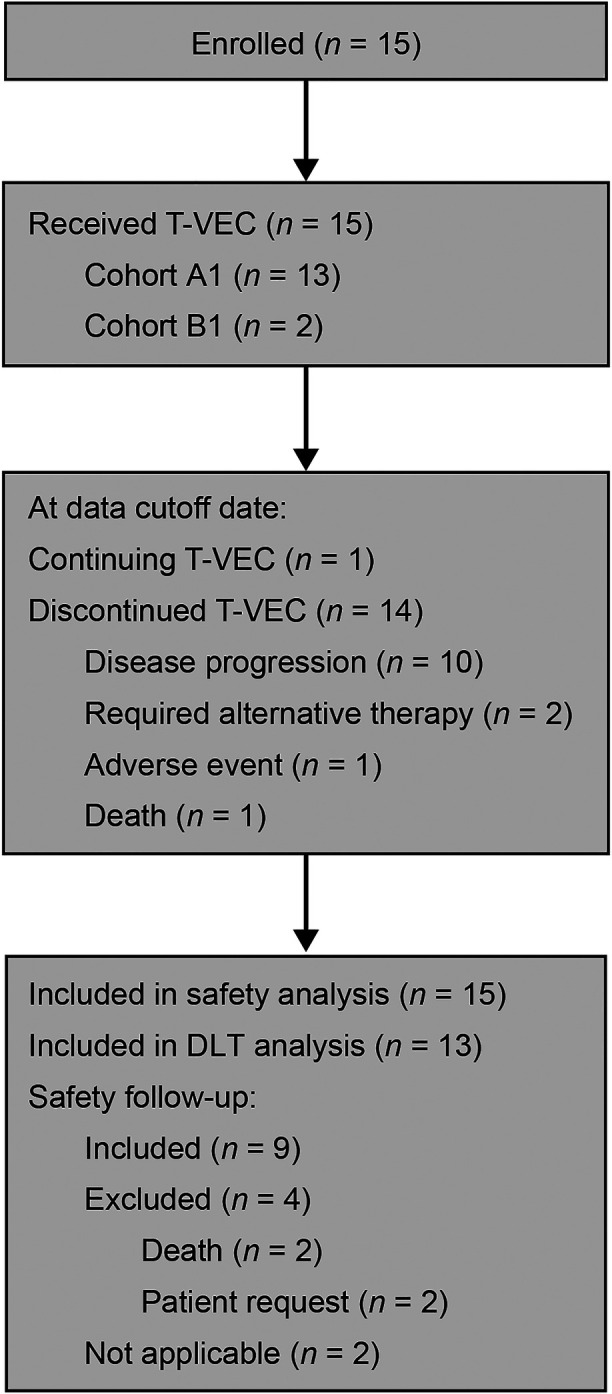
Patient disposition. DLT, dose-limiting toxicity; T-VEC, talimogene laherparepvec. A total of 15 patients were enrolled into two cohorts stratified by age: cohort A1 (*n* = 13), patients aged 12 to ≤21 years of age; and cohort B1 (*n* = 2), patients aged 2 to <12 years of age. All patients received T-VEC during the study. At the time of data cutoff, 14 patients (93.3%) had discontinued the investigational product. Reasons included disease progression (10 patients), requirement for alternative therapy (two patients), adverse event (one patient), or death (one patient). The global end of the study occurred in November 2022.

Between August 2017 and January 2022, 15 pediatric patients across Europe and North America were assigned to receive T-VEC (Supplementary Table S1). Table 1 summarizes the patients' baseline characteristics. Patients were enrolled into two cohorts based on age: cohort A1 (*N* = 13) aged 12 to ≤21 (soft-tissue sarcoma, *n* = 7; bone tumor, *n* = 3; neuroblastoma, *n* = 1; nasopharyngeal carcinoma, *n* = 1; and melanoma, *n* = 1) and cohort B1 (*N* = 2) aged 2 to <12 (melanoma, *n* = 2) (Figure 1 and Table 1). Five patients had visceral disease at baseline in cohort A1 and one patient had visceral disease in cohort B1. The sum of the largest diameters of target lesions was less than 200 mm for 12 patients in cohort A1 and for both patients in cohort B1. The overall median (range) age was 14 (7, 21) years. Ten patients were male, five were female, and most were White. Seven patients had HSV-1–positive serostatus at baseline. Overall, 14 patients had received prior systemic anticancer therapy (three patients received two lines of prior therapy, six patients received three lines of prior therapy, one patient received four lines of prior, and three patients received more than four lines of prior therapy). Thirteen patients had received prior surgery for malignancy. Eleven patients had received prior radiotherapy for malignancy. At the time of data cutoff, one patient was on treatment and 14 patients had discontinued the investigational product. Discontinuation was due to death (*n* = 1), disease progression (*n* = 10), requirement for alternative therapy (*n* = 2), and adverse event (*n* = 1).

**Table 1:** Baseline patient demographics and characteristics.

Characteristic	Cohort A1	Cohort B1	Total
	(*N* = 13)	(*N* = 2)	(*N* = 15)
Sex			
Male	8	2	10
Female	5	-	5
Median age, years (range)	14 (12, 21)	9 (7, 11)	14 (7, 21)
Ethnicity			
Not Hispanic/Latino	10	1	11
Hispanic/Latino	2	1	3
Unknown	1	-	1
Race			
White	9	2	11
American Indian or Alaska Native	1	-	1
Other	3	-	3
Positive HSV-1 status at baseline	7	-	7
Lansky play performance status			
NA^a^	13	-	13
>70%	-	2	2
Karnofsky play performance status			
>70%	11	-	11
70%	2	-	2
NA^b^	-	2	2
Prior surgery	11	2	13
Prior radiotherapy	10	1	11
Prior anticancer therapy	12^c^	2	14
Maximum lines of prior therapy			
None	1	0	1
1	1	0	1
2	2	1	3
3	6	0	6
4	1	0	1
>4	2	1	3
Sum of the largest diameters of target lesions (mm)			
0 to <200	12	2	14
400 to <500	1	-	1
Visceral disease at baseline			
Yes	5	1	6
No	8	1	9
Tumor type			
Soft tissue sarcoma	7	-	7
Non-rhabdomyosarcoma soft tissue sarcoma	6	-	6
Rhabdomyosarcoma	1	-	1
Bone tumor	3	-	3
Osteosarcoma	2	-	2
Ewing sarcoma	1	-	1
Neuroblastoma	1	-	1
Nasopharyngeal carcinoma	1	-	1
Melanoma	1	2	3

Data represent number of patients, unless otherwise specified; HSV-1, herpes simplex virus type-1; NA, not applicable.

^a^ Lansky play performance status is applicable to children aged <12 years.

^b^ Karnofsky play performance status is applicable to patients aged 12 to ≤21 years.

^c^ One patient had no previous anticancer therapy. This patient had fibromyxoid sarcoma with recurrent metastatic diseases and failed standard-of-care surgery + radiation. Procedure of complete surgical resection of all gross diseases includes all surgeries that had no residual disease described by the investigator. Patients whose initial disease stage data were missing were excluded from the summary of initial disease stage.

Initially, three patients were enrolled in cohort A1 (patients aged 12 to ≤21) and treated at 100% of the recommended adult dose of T-VEC (Supplementary Figures S1–2). After reviewing the safety data for these initial patients, no DLTs were reported and the DLRT opened cohort B1 (patients aged 2 to <12) for enrollment and treatment at the same dose level.

### Exposure

Patients received T-VEC for a median (range) of 5.1 (0.1, 39.4) weeks, shown according to tumor type in Supplementary Figure S3. The mean (standard deviation [SD]) volume for the first injection was 3.5 (0.8) ml of 10^6^ PFU/ml T-VEC. For all other doses, the mean (SD) volume of injection was 3.7 (0.6) ml of 10^8^ PFU/ml T-VEC.

### Primary endpoint analysis

The DLT analysis set included 13 patients (11 patients from cohort A1 and two from cohort B1) in whom no DLTs were observed during the DLT evaluation period.

### Safety

No patients had a herpetic event and all 15 patients experienced at least one TEAE. The most frequently reported any-grade TEAEs (≥20% of patients) were pyrexia (*n* = 11), vomiting (*n* = 7), and headache (*n* = 6) (Table 2). Grade ≥3 TEAEs occurred in eight patients. The most frequently reported grade 3 TEAE was anaemia (*n* = 2). No grade 4 or 5 TEAEs were reported in any cohort. One patient had a grade 2 TEAE of wound dehiscence leading to discontinuation of T-VEC. The event occurred after the third dose of T-VEC, proximal to the site of the injection, and developed into an ulcer after 1 week. A lesion swab test of the ulcer was positive for HSV-1. Possible causes included chronic radiation dermatitis, but T-VEC could not be excluded as a contributing factor.

**Table 2:** TEAEs by preferred term and grade in the safety analysis set.

	Cohort A1		Cohort B1		Total	
	(*N* = 13)		(*N* = 2)		(*N* = 15)	
TEAE	Any grade	Grade ≥3	Any grade	Grade ≥3	Any grade	Grade ≥3
Pyrexia	9	-	2	-	11	-
Vomiting	6	-	1	-	7	-
Headache	5	-	1	-	6	-
Pain in extremity	4	-	1	-	5	-
Fatigue	4	-	-	-	4	-
Nausea	3	1	1	-	4	1
Anaemia	3	2	-	-	3	2
Influenza like illness	3	-	-	-	3	-
Gamma-glutamyltransferase increased	2	-	1	-	3	-
Arthralgia	2	-	-	-	2	-
Asthenia	2	1	-	-	2	1
Back pain	2	-	-	-	2	-
Constipation	2	-	-	-	2	-
Cough	2	-	-	-	2	-
Diarrhoea	2	-	-	-	2	-
Influenza	2	-	-	-	2	-
Injection site pain	2	-	-	-	2	-
Paraesthesia	2	-	-	-	2	-
Sinus tachycardia	2	-	-	-	2	-
Abdominal pain	1	-	-	-	1	-
Abdominal pain upper	1	-	-	-	1	-
Autoimmune hypothyroidism	1	-	-	-	1	-
Chest pain	1	-	-	-	1	-
Chills	1	-	-	-	1	-
Coagulopathy	1	-	-	-	1	-
Decreased appetite	1	-	-	-	1	-
Deep vein thrombosis	1	-	-	-	1	-
Dehydration	1	-	-	-	1	-
Drug hypersensitivity	1	-	-	-	1	-
Dry mouth	1	-	-	-	1	-
Dyspnoea exertional	1	-	-	-	1	-
Embolism	1	-	-	-	1	-
Erythema	1	-	-	-	1	-
Face oedema	1	-	-	-	1	-
Haematuria	1	-	-	-	1	-
Heat cramps	1	-	-	-	1	-
Hot flush	1	-	-	-	1	-
Hypokalaemia	1	1	-	-	1	1
Hyponatraemia	1	1	-	-	1	1
Injection site inflammation	1	-	-	-	1	-
Joint contracture	1	-	-	-	1	-
Malaise	1	-	-	-	1	-
Mastitis	1	-	-	-	1	-
Muscle spasms	1	-	-	-	1	-
Musculoskeletal chest pain	1	1	-	-	1	1
Myalgia	1	-	-	-	1	-
Nail infection	1	1	-	-	1	1
Nasal congestion	1	-	-	-	1	-
Neuralgia	1	-	-	-	1	-
Oedema peripheral	1	-	-	-	1	-
Photosensitivity reaction	1	-	-	-	1	-
Presyncope	1	-	-	-	1	-
Procedural pain	1	-	-	-	1	-
Proteinuria	1	-	-	-	1	-
Pruritus	1	-	-	-	1	-
Pulmonary embolism	1	1	-	-	1	1
Rhinitis	1	-	-	-	1	-
Rhinorrhoea	1	-	-	-	1	-
Skin ulcer	1	1	-	-	1	1
Tachycardia	1	-	-	-	1	-
Upper respiratory tract infection	1	-	-	-	1	-
Vascular device infection	1	1	-	-	1	1
Wheezing	1	-	-	-	1	-
Wound dehiscence	1	-	-	-	1	-
Haemorrhage	-	-	1	-	1	-
Musculoskeletal pain	-	-	1	-	1	-

Data represent number of patients. Safety analysis set includes all patients who received at least one dose of T-VEC. TEAEs are defined as any event occurring after the first dose through 30 days after the last dose of T-VEC. Adverse events were coded using MedDRA version 24.1. MedDRA, Medical Dictionary for Regulatory Activities; TEAE, treatment-emergent adverse event; T-VEC, talimogene laherparepvec.

Treatment-emergent serious adverse events occurred in four patients (musculoskeletal chest pain, nausea, pulmonary embolism, skin ulcer, and vascular device infection; *n* = 1 each). Overall, 13 patients experienced treatment-related TEAEs. Three patients had grade 3 or 4 treatment-related TEAEs (asthenia, pulmonary embolism, hyponatraemia, and skin ulcer; *n* = 1 each) (Table 3). Fatal disease–related adverse events (osteosarcoma metastatic and hypovolemic shock) were reported in two patients.

**Table 3:** Grade 3 or 4 treatment-related TEAEs by preferred term in the safety analysis set.

Grade 3 or 4 Treatment-Related TEAEs	Cohort A1	Cohort B1	Total
	(*N* = 13)	(*N* = 2)	(*N* = 15)
Asthenia	1	-	1
Hyponatraemia	1	-	1
Pulmonary embolism	1	-	1
Skin ulcer	1	-	1

Data are presented as number of patients. Safety analysis set includes all patients who received at least one dose of T-VEC. Treatment-related TEAEs are defined as any event occurring after the first dose through 30 days after the last dose of T-VEC. Adverse events were coded using MedDRA version 24.1. Severity of each adverse event was graded using CTCAE version 4.0. CTCAE, Common Terminology Criteria for Adverse Events; MedDRA, Medical Dictionary for Regulatory Activities; TEAE, treatment-emergent adverse event; T-VEC, talimogene laherparepvec.

### Response to therapy

Efficacy results by cohort are summarized in Table 4. The ORR per modified irRC-RECIST was 0% (95% CI: 0.00, 21.80). All patients were assessed for best overall responses: no patient had a complete or partial response, three had stable disease, five had PD, and seven were either unevaluable (*n* = 4) or a response evaluation was missing (*n* = 3). The three patients with stable disease received treatment for 16, 38, and 40 weeks. The median PFS was 1.7 (95% CI: 1.3, 6.9) months in cohort A1 and 6.4 (95% CI: 1.6, not estimable [NE]) months in cohort B1 (Supplementary Figure S4). Overall, the 6- and 9-month PFS estimates were 31.7 (95% CI: 9.9, 56.5) and 15.9 (95% CI: 2.6, 39.7), respectively.

**Table 4:** Best overall response per modified irRC-RECIST criteria per investigator in the safety analysis set.

Best Overall Response	Cohort A1	Cohort B1	Total
	(*N* = 13)	(*N* = 2)	(*N* = 15)
Complete response	-	-	-
Partial response	-	-	-
Stable disease	2	1	3
Progressive disease	4	1	5
Unevaluable^a^	4	-	4
Missing^b^	3	-	3
Objective response rate (CR/PR)	-	-	-
95% CI^c^	(0.00, 24.71)	(0.00, 84.19)	(0.00, 21.80)

Data are presented as number of patients. Objective response rate is defined as the incidence of a best overall response of CR or PR per modified irRC-RECIST version 1.1 response criteria in the safety analysis set. Patients who do not have any follow-up tumor assessments are regarded as non-responders. Safety analysis set includes all patients who received at least one dose of T-VEC. CR, complete response; irRC-RECIST, immune-related response criteria simulating the Response Evaluation Criteria in Solid Tumors; PR, partial response; T-VEC, talimogene laherparepvec.

^a^ Based on a clinical review of the data, four patients had a best overall response of unevaluable due to incomplete assessment of disease (*n* = 2), an initial progressive disease with no confirmation of progressive disease prior to subsequent anticancer therapy (*n* = 1), and a partial resection prior to observation of progression or response (*n* = 1).

^b^ Three patients had a best overall response of missing due to no post baseline radiographic disease assessments recorded prior to analysis data cutoff; two of these patients were reported to have a non-radiographic progression and received new antitumor therapy prior to the first planned radiographic assessment time.

^c^ Binomial proportion with exact 95% CI.

### T-VEC clearance

T-VEC DNA was detected by qPCR analysis in the blood of all patients (*n* = 15) throughout the study, with peak detection in the blood in 14 patients at week 4. Among the 15 patients, 14 had detectable T-VEC DNA in swab samples taken during the study, primarily from the surface of injected lesions. Two of seven patients had detectable T-VEC DNA swab samples taken at the time of safety follow-up (surface of injected lesions). No patients had detectable T-VEC DNA in the urine.

### Unintended exposure to T-VEC

Unintended T-VEC exposure was monitored throughout the study treatment as well as up to 37 days after the last dose of T-VEC. Two patients' close contacts and two healthcare providers had signs/symptoms of herpes infection. These individuals reported a cold sore/fever blister event after a patient on the study received T-VEC, but no direct T-VEC exposure was reported. Swab tests were performed for healthcare professionals and were negative for T-VEC. The symptoms resolved without complications in all cases.

## Discussion

There remains an unmet need for novel therapeutic strategies to treat pediatric patients with refractory solid tumors. This is the first study to assess the safety and tolerability of the oncolytic virus T-VEC in pediatric patients with extracranial solid tumors. The present study met its primary objective of determining the safety and tolerability of T-VEC, and we report that treatment with T-VEC at adult doses and volumes is safe and tolerable in pediatric patients aged 2 to ≤21 years as assessed by no reported DLTs. The safety profile and adverse events were consistent with T-VEC's mechanism of action and the known safety profile of T-VEC reported in studies with adult patients. There were no reported antitumor responses in the different pediatric solid tumors evaluated. The study was terminated early after discussion with the EMA.

Safety results reported in the present study were consistent with reports from adult clinical trials of T-VEC monotherapy or in combinations with other targeted agents (23, 24). Amongst the most common adverse events in adults treated with T-VEC are flu-related symptoms, such as fatigue (50.3%), chills (48.6%), and pyrexia (42.8%) (14). Similarly, in our study, the most common adverse events were also flu-related symptoms, such as pyrexia, vomiting, and headache, and T-VEC treatment was deemed tolerable and safe at the standard dose in these pediatric patients. Furthermore, the rate of HSV-1 seropositivity aligns with that reported in the literature for this age group and is also lower than that observed in adults, as is expected (17). Based on previous studies conducted in adult patients, we did not predict any impact of the baseline HSV-1 status on the results of this study except for an increase in flu-like symptoms in patients who were HSV-1 negative at baseline (14, 23, 24). T-VEC DNA was detected in the blood of all patients during treatment and not in the urine, comparable with previous reports that have demonstrated detectable T-VEC DNA in the blood of adult patients (98.3%) and at low levels in the urine across treatment (25).

Furthermore, the results of the present study correlate with the expected T-VEC DNA clearance mechanism and seroconversion pathway for the adult dose schedule and demonstrate a similar clearance mechanism in the pediatric population.

The lack of responses in the present study could be related to the tumor microenvironment and immunological profile of the diverse tumor types evaluated in this study. There is no information to suggest that the mechanisms of action of T-VEC (including viral replication in injected tumors and immune response against tumor antigens) would differ in children and adolescents compared with adults, although the efficacy of T-VEC has been shown to vary amongst tumor types (24, 26). The OPTiM trial comparing T-VEC and GM-CSF in adult patients with melanoma demonstrated a durable response rate of 16.3% vs. 2.1% and an ORR of 26.4% vs. 5.7%, respectively (14). Patients with Merkel cell carcinoma treated with T-VEC showed a median PFS of greater than 16 months, and patients with advanced pancreatic tumors have also demonstrated durable clinical responses (27, 28). Other adult non-melanoma studies have not demonstrated objective responses and have included patients with breast cancer, gastrointestinal adenocarcinoma, or head and neck squamous cell carcinoma (24). No objective responses were observed in the heavily pretreated population of the present study, although two patients with soft tissue carcinoma received treatment beyond 6 months, which may represent some disease control.

Like T-VEC, other intratumoral oncolytic therapies given as single agents have not successfully demonstrated objective responses in pediatric solid tumors, such as HSV1716 (12), NTX-010 (8), and Reolysin (9). Efforts continue for agents such as DNX-2401 and HSV-1 G207 that are being explored alone or in combination with radiotherapy in patients with glioma and have shown promising phase 1 results (29). This highlights that the tumor type and its microenvironment may impact the response to oncolytic viral intervention. Further research is required to assess the relationship between virus replication, antitumor immunity, and the microenvironment. Intralesional injection of T-VEC has been shown previously to impact the tumor microenvironment by inducing an oncolytic immune-mediated effect, as shown by increased circulating CD4^+^ and CD8^+^ T cells (30). However, the objectives of the present study did not include the evaluation of biomarkers to assess the impact of T-VEC on the immune system.

Both the prevalence and type of cancer differs between pediatric and adult populations, making it difficult to evaluate if treatments that are effective in the adult population are also effective in the pediatric population (31). Critically, the immune responses in the pediatric and adult populations may differ, and this could impact the time to treatment response for patients with relapsed solid tumors (4, 5). Pediatric patients typically enter phase 1 trials at a later stage of their disease, often with relapsed solid tumors and high tumor burdens, leading to a median OS of just 6.3 (95% CI: 5.2, 7.4) months (32). In comparison, the OPTiM study included adult patients with advanced melanoma, and despite the advanced stage of their disease, these patients had a median OS of 23.3 (95% CI: 19.5, 29.6) months with T-VEC (14, 33). The disparity between the OS of pediatric and adult populations emphasizes that the short therapeutic window of aggressive pediatric cancers may be a critical factor affecting pediatric trial outcomes in response to T-VEC and other immunotherapies.

One limitation of this study was the challenge of patient recruitment due to the exclusion of pediatric patients with visceral lesions or CNS tumors. Therefore, only patients with cutaneous/subcutaneous/soft tissue/lymph tumors, including those located in the limbs, were eligible. The eligibility criteria of the present study was revised to include patients with a history of previously treated brain metastasis with radiographic evidence of improvement or no evidence of disease progression before screening. Second, the tumors of eligible patients needed to be amenable to intratumoral injection, which limited the applicable tumor types. Next, the present study did not enroll patients younger than 2 years of age and included only two patients younger than 12 years of age. Therefore, the results may not apply to this younger age group. Finally, the sample size of the current study was small (*N* = 15), which limits interpretation of the results.

Although no objective responses were observed in these late-stage/refractory patients, the tolerability of T-VEC as assessed by no reported DLTs introduces an opportunity for the assessment of other targeted combination strategies in pediatric patients with early-stage or advanced non-CNS tumors. Combination therapies might be more effective and are currently being evaluated in adult melanoma patients (34).

## Data Availability

The datasets presented in this article are not readily available, although qualified researchers may request data from Amgen clinical studies. Complete details are available at the following web address: http://www.amgen.com/datasharing. Requests to access the datasets should be directed to http://www.amgen.com/datasharing.

## Supplementary material

SUPPLEMENTARY FIGURE S1

**Study Design and Treatment Schema.** D, day; DLRT, dose-level review team; DLT, dose-limiting toxicity; EOS, end of study; EOT, end of treatment; FU, follow-up; irRC-RECIST, immune-related response criteria simulating Response Evaluation Criteria in Solid Tumors; PFU, plaque-forming unit; Q2W, once every 2 weeks; T-VEC, talimogene laherparepvec; y, years of age. ^1^Enrollment began with cohort A1. The DLT evaluation period was 35 days from the first dose of T-VEC. The DLRT reviewed the safety data of the first three patients in the older cohort A1 to decide if the younger age cohort B1 could be opened for enrollment. If none of the first three DLT-evaluable patients in cohort A1 experienced a DLT, then cohort B1 was opened for enrollment and treatment carried out at the same dose level. If a DLT occurred in the first three DLT-evaluable patients in cohort A1, B1 was not opened until a DLT rate <33% was observed with at least six DLT-evaluable patients in cohort A1. ^2^The initial dose administered (dose level 1) was up to 4.0 ml of 10^6 ^PFU/ml, followed by a dose of up to 4.0 ml of 10^8^ PFU/ml 21 (+3) days later. Subsequent doses of up to 4.0 ml of 10^8 ^PFU/ml were administered every 14 (±3) days thereafter. ^3^If the observed DLT rate was ≥33% in a cohort or if the DLRT deemed the dose intolerable in a cohort in the DLT evaluation period, then dose de-escalation or discontinuation of treatment with subsequent enrollment stop for that cohort occurred. ^4^If dose de-escalation was needed and if permissible based on the incidence of DLTs, a minimum of six DLT-evaluable patients were enrolled and treated at a lower dose level (dose level −1). ^5^Treatment continued until a complete response, disappearance of all injectable tumors, confirmed progressive disease per modified irRC-RECIST, intolerance of study treatment, 24 months from the date of the first dose of T-VEC, or need for alternative anticancer therapy, whichever occurred first. ^6^If permissible based on the incidence of DLTs. ^7^Patients were followed up for survival and use of subsequent anticancer therapies every 12 weeks (±28 days) from the safety follow-up visit until death, consent withdrawal, or up to approximately 24 months after the last patient was enrolled, whichever occurred first.

SUPPLEMENTARY FIGURE S2

**Dose De-escalation Rules.** DLRT, dose-level review team; DLT, dose-limiting toxicity; PFU, plaque-forming unit; T-VEC, talimogene laherparepvec; y, years of age. ^1^Enrollment began with cohort A1. The DLT evaluation period was 35 days from the first dose of T-VEC. The DLRT reviewed the safety data of the first three patients in the older cohort A1 to decide if the younger age cohort B1 could be opened for enrollment. If none of the first three DLT-evaluable patients in cohort A1 experienced a DLT, then cohort B1 was opened for enrollment and treatment carried out at the same dose level. If a DLT occurred in the first three DLT-evaluable patients in cohort A1, B1 was not opened until a DLT rate < 33% was observed with at least six DLT-evaluable patients in cohort A1. ^2^The initial dose administered (dose level 1) was up to 4.0 ml of 10^6^ PFU/ml, followed by a dose of up to 4.0 ml of 10^8 ^PFU/ml 21 (+3) days later. Subsequent doses of up to 4.0 ml of 10^8 ^PFU/ml were administered every 14 (±3) days thereafter. ^3^If the observed DLT rate was ≥33% in a cohort or if the DLRT deemed the dose intolerable in a cohort in the DLT evaluation period, then dose de-escalation or discontinuation of treatment with subsequent enrollment stop for that cohort occurred. ^4^If dose de-escalation was needed and if permissible based on the incidence of DLTs, a minimum of six DLT-evaluable patients were enrolled and treated at a lower dose level (dose level −1). ^5^If permissible based on the incidence of DLTs.

SUPPLEMENTARY FIGURE S3

**Duration of Therapy According to the Primary Tumor Type of Patients in the Safety Analysis Set**. NRSTS, non-rhabdomyosarcoma soft-tissue sarcoma; PNET, primitive neuroectodermal tumor; RMS, rhabdomyosarcoma; T-VEC, talimogene laherparepvec. Median indicates median duration (weeks). Treatment duration (weeks) = (T-VEC last dose date – T-VEC first dose date + 1)/7, where the injected volume >0 ml for both the dates. Subgroup of different tumor types at baseline is distinguished by different colors: osteosarcoma and Ewing sarcoma/PNET into malignant bone tumor (as one color); neuroblastoma and ganglioneuroblastoma into neuroblastoma (as one color); nasopharyngeal carcinoma into other malignant solid tumor (as one color); nodular melanoma and unclassifiable into skin, melanoma (as one color); and RMS and NRSTS into soft tissue sarcoma (as one color).

SUPPLEMENTARY FIGURE S4

**PFS per irRC-RECIST Criteria Per Investigator.** CI, confidence interval; irRC-RECIST, immune-related response criteria simulating Response Evaluation Criteria in Solid Tumors; *N*, number of patients in the safety analysis set; NE, not estimable; PD, progressive disease; PFS, progression-free survival; T-VEC, talimogene laherparepvec. PFS per modified irRC-RECIST version 1.1 is defined as the interval from the first dose to the earlier of PD per modified irRC-RECIST or death from any cause; otherwise, PFS was censored at the last evaluable tumor assessment. The initial date of PD was the PFS date when it was consecutively confirmed. The safety analysis set included all patients who received at least one dose of T-VEC. Patients with no event or no evaluable tumor assessments were censored on study day 1. Censoring is indicated by a vertical line.

## References

[1] Steliarova-Foucher E Stiller C Kaatsch P Berrino F Coebergh JW Lacour B Geographical patterns and time trends of cancer incidence and survival among children and adolescents in Europe since the 1970s (the ACCISproject): an epidemiological study. Lancet. (2004) 364(9451):2097–105. 10.1016/S0140-6736(04)17550-8 15589307

[2] Pui CH Gajjar AJ Kane JR Qaddoumi IA Pappo AS. Challenging issues in pediatric oncology. Nat Rev Clin Oncol. (2011) 8(9):540–9. 10.1038/nrclinonc.2011.95 21709698 PMC3234106

[3] Vogelstein B Papadopoulos N Velculescu VE Zhou S Diaz LA Jr. Kinzler KW. Cancer genome landscapes. Science. (2013) 339(6127):1546–58. 10.1126/science.1235122 23539594 PMC3749880

[4] Terry RL Meyran D Ziegler DS Haber M Ekert PG Trapani JA Immune profiling of pediatric solid tumors. J Clin Invest. (2020) 130(7):3391–402. 10.1172/JCI137181 32538896 PMC7324195

[5] Long AH Morgenstern DA Leruste A Bourdeaut F Davis KL. Checkpoint immunotherapy in pediatrics: here, gone, and back again. Am Soc Clin Oncol Educ Book. (2022) 42:1–14. 10.1200/EDBK_349799 35580293

[6] Pearson ADJ Rossig C Lesa G Diede SJ Weiner S Anderson J ACCELERATE And European medicines agency paediatric strategy forum for medicinal product development of checkpoint inhibitors for use in combination therapy in paediatric patients. Eur J Cancer. (2020) 127:52–66. 10.1016/j.ejca.2019.12.029 31986450

[7] Kaufman HL Kohlhapp FJ Zloza A. Oncolytic viruses: a new class of immunotherapy drugs. Nat Rev Drug Discov. (2015) 14(9):642–62. 10.1038/nrd4663 26323545 PMC7097180

[8] Burke MJ Ahern C Weigel BJ Poirier JT Rudin CM Chen Y Phase I trial of seneca valley virus (NTX-010) in children with relapsed/refractory solid tumors: a report of the children's oncology group. Pediatr Blood Cancer. (2015) 62(5):743–50. 10.1002/pbc.25269 25307519 PMC4376652

[9] Kolb EA Sampson V Stabley D Walter A Sol-Church K Cripe T A phase I trial and viral clearance study of reovirus (reolysin) in children with relapsed or refractory extra-cranial solid tumors: a children's oncology group phase I consortium report. Pediatr Blood Cancer. (2015) 62(5):751–8. 10.1002/pbc.25464 25728527 PMC4376570

[10] Cripe TP Ngo MC Geller JI Louis CU Currier MA Racadio JM Phase 1 study of intratumoral Pexa-vec (JX-594), an oncolytic and immunotherapeutic vaccinia virus, in pediatric cancer patients. Mol Ther. (2015) 23(3):602–8. 10.1038/mt.2014.243 25531693 PMC4351466

[11] Ruano D Lopez-Martin JA Moreno L Lassaletta A Bautista F Andion M First-in-Human, first-in-child trial of autologous MSCs carrying the oncolytic virus icovir-5 in patients with advanced tumors. Mol Ther. (2020) 28(4):1033–42. 10.1016/j.ymthe.2020.01.019 32053771 PMC7132606

[12] Streby KA Geller JI Currier MA Warren PS Racadio JM Towbin AJ Intratumoral injection of HSV1716, an oncolytic herpes virus, is safe and shows evidence of immune response and viral replication in young cancer patients. Clin Cancer Res. (2017) 23(14):3566–74. 10.1158/1078-0432.CCR-16-2900 28495911 PMC10546618

[13] Liu BL Robinson M Han ZQ Branston RH English C Reay P ICP34.5 Deleted herpes simplex virus with enhanced oncolytic, immune stimulating, and anti-tumour properties. Gene Ther. (2003) 10(4):292–303. 10.1038/sj.gt.3301885 12595888

[14] Andtbacka RH Kaufman HL Collichio F Amatruda T Senzer N Chesney J Talimogene laherparepvec improves durable response rate in patients with advanced melanoma. J Clin Oncol. (2015) 33(25):2780–8. 10.1200/JCO.2014.58.3377 26014293

[15] Zuch de Zafra C Beltran P. Nonclinical efficacy of talimogene laherparepvec in pediatric tumor types. 2017 American society of clinical oncology (ASCO) annual meeting; June 2-6, 2017

[16] Waters AM Friedman GK Ring EK Beierle EA. Oncolytic virotherapy for pediatric malignancies: future prospects. Oncolytic Virother. (2016) 5:73–80. 10.2147/OV.S96932 27579298 PMC4996251

[17] Smith JS Robinson NJ. Age-specific prevalence of infection with herpes simplex virus types 2 and 1: a global review. J Infect Dis. (2002) 186(Suppl 1):S3–28. 10.1086/343739 12353183

[18] Storer BE. Design and analysis of phase I clinical trials. Biometrics. (1989) 45(3):925–37. 10.2307/2531693 2790129

[19] Nishino M Gargano M Suda M Ramaiya NH Hodi FS. Optimizing immune-related tumor response assessment: does reducing the number of lesions impact response assessment in melanoma patients treated with ipilimumab? J Immunother Cancer. (2014) 2:17. 10.1186/2051-1426-2-17 24991412 PMC4077549

[20] IMLYGIC® (talimogene laherparepvec) Safety Data Sheet. Revision Number: 11. Date Issued: February 20. https://www.msds.amgen.com/-/media/Themes/Amgen/msds-amgen-com/msds-amgen-com/documents/imlygicsds.pdf Accessed 03 April 2023.

[21] Ferrucci PF Pala L Conforti F Cocorocchio E. Talimogene laherparepvec (T-VEC): an intralesional cancer immunotherapy for advanced melanoma. Cancers (Basel). (2021) 13(6):1383. 10.3390/cancers13061383 PMC8003308 33803762

[22] Rehman H Silk AW Kane MP Kaufman HL. Into the clinic: talimogene laherparepvec (T-VEC), a first-in-class intratumoral oncolytic viral therapy. J Immunother Cancer. (2016) 4(1):53. 10.1186/s40425-016-0158-5 27660707 PMC5029010

[23] Puzanov I Milhem MM Minor D Hamid O Li A Chen L Talimogene laherparepvec in combination with ipilimumab in previously untreated, unresectable stage IIIB-IV melanoma. J Clin Oncol. (2016) 34(22):2619–26. 10.1200/JCO.2016.67.1529 27298410 PMC7189507

[24] Hu JC Coffin RS Davis CJ Graham NJ Groves N Guest PJ A phase I study of OncoVEXGM-CSF, a second-generation oncolytic herpes simplex virus expressing granulocyte macrophage colony-stimulating factor. Clin Cancer Res. (2006) 12(22):6737–47. 10.1158/1078-0432.CCR-06-0759 17121894

[25] Andtbacka RHI Amatruda T Nemunaitis J Zager JS Walker J Chesney JA Biodistribution, shedding, and transmissibility of the oncolytic virus talimogene laherparepvec in patients with melanoma. EBioMedicine. (2019) 47:89–97. 10.1016/j.ebiom.2019.07.066 31409575 PMC6796514

[26] Salloum A Koblinski J Bazzi N Zeitouni NC. Talimogene laherparepvec in non-melanoma cancers. J Clin Aesthet Dermatol. (2021) 14(11):18–25. 34980955 PMC8675341

[27] Westbrook BC Norwood TG Terry NLJ McKee SB Conry RM. Talimogene laherparepvec induces durable response of regionally advanced Merkel cell carcinoma in 4 consecutive patients. JAAD Case Rep. (2019) 5(9):782–6. 10.1016/j.jdcr.2019.06.034 31516997 PMC6728723

[28] Chang KJ Senzer NN Binmoeller K Goldsweig H Coffin R. Phase I dose-escalation study of talimogene laherparepvec (T-VEC) for advanced pancreatic cancer (ca). J Clin Oncol. (2012) 30(15_suppl):e14546-e. 10.1200/jco.2012.30.15_suppl.e14546

[29] Gallego Perez-Larraya J Garcia-Moure M Labiano S Patino-Garcia A Dobbs J Gonzalez-Huarriz M Oncolytic DNX-2401 virus for pediatric diffuse intrinsic pontine glioma. N Engl J Med. (2022) 386(26):2471–81. 10.1056/NEJMoa2202028 35767439

[30] Hughes T Coffin RS Lilley CE Ponce R Kaufman HL. Critical analysis of an oncolytic herpesvirus encoding granulocyte-macrophage colony stimulating factor for the treatment of malignant melanoma. Oncolytic Virother. (2014) 3:11–20. 10.2147/OV.S36701. eCollection 2014 27512660 PMC4918360

[31] Siegel RL Miller KD Fuchs HE Jemal A. Cancer statistics, 2022. CA Cancer J Clin. (2022) 72(1):7–33. 10.3322/caac.21708 35020204

[32] Carceller F Bautista FJ Jimenez I Hladun-Alvaro R Giraud C Bergamaschi L Prognostic factors of overall survival in children and adolescents enrolled in dose-finding trials in Europe: an innovative therapies for children with cancer study. Eur J Cancer. (2016) 67:130–40. 10.1016/j.ejca.2016.08.008 27662616

[33] Andtbacka RH Ross M Puzanov I Milhem M Collichio F Delman KA Patterns of clinical response with talimogene laherparepvec (T-VEC) in patients with melanoma treated in the OPTiM phase III clinical trial. Ann Surg Oncol. (2016) 23(13):4169–77. 10.1245/s10434-016-5286-0 27342831 PMC5090012

[34] Puzanov I Chesney J Collichio F Singh P Milhem M Glaspy J 433 Talimogene laherparepvec (T-VEC) in combination with ipilimumab (IPI) versus IPI alone for advanced melanoma: 4-year interim analysis of a randomized, open-label, phase 2 trial. J Immunother Cancer. (2020) 8(Suppl 3):A263–A4. 10.1136/jitc-2020-SITC2020.0433 PMC10163510 37142291

