# Supplementary material for: A phase 1, first-in-child, multicenter study to evaluate the safety and efficacy of the oncolytic herpes virus talimogene laherparepvec in pediatric patients with advanced solid tumors
Source: Front Pediatr. 2023 May 24;11:1183295. doi: 10.3389/fped.2023.1183295 (PMC10244735; doi:10.3389/fped.2023.1183295)
Supplement: Supplementary file 1 [file Table1.pdf]

Supplementary Table 1. List of Institutional Review Board (IRB) or Independent Ethics Committee (IEC) for all sites that enrolled patients

Site Number	Site Name	IRB/IEC Name	IRB/IEC Address	Regional IRB/IEC Name	Regional IRB/IEC Address
13001	Universitair Ziekenhuis Gent	Universitair Ziekenhuis Gent - Ethisch Comite	Corneel Heymanslaan 10, Ingang 75, tweede verdieping, Gent, 9000, Belgium	Universitair Ziekenhuis Gent - Ethisch Comite	Corneel Heymanslaan 10, Ingang 75, tweede verdieping, Gent, 9000, Belgium
16001	Centre Hospitalier Universitaire Sainte Justine	Comité d'éthique de la recherche du Centre Hospitalier Universitaire Sainte-Justine	3175 Cote Sainte-Catherine, Room A-558, Montreal, QC, H3T 1C5, Canada		
25001	Centre Hospitalier Universitaire de Marseille - Hopital de la Timone	Comite de Protection des Personnes Sud-Est III - Groupement hospitalier est	59 boulevard Pinel, Batiment Pinel, Bron, 69500, France	Comite de Protection des Personnes Sud-Est III - roupement hospitalier est	59 boulevard Pinel, Batiment Pinel, Bron, 69500, France
25004	Institut Curie	Comite de Protection des Personnes Sud-Est III - Groupement hospitalier est	59 boulevard Pinel, Batiment Pinel, Bron, 69500, France	Comite de Protection des Personnes Sud-Est III - roupement hospitalier est	59 boulevard Pinel, Batiment Pinel, Bron, 69500, France
58001	Hospital Universitario Infantil Niño Jesus	CEIC Hospital Universitario Niño Jesus	Avenida de Menendez Pelayo 65, Madrid, Madrid, 28009, Spain	CEIC Hospital Universitari Vall d Hebron	Passeig de la Vall d Hebron 119-129, Institut de Recerca Ed Maternoinfantil Pl 13, Barcelona, Cataluña, 08035, Spain

58002	Hospital Sant Joan de Deu	CEIC Fundacio Sant Joan de Deu	Carrer de Santa Rosa 39-57, Planta 3, Esplugues de Llobregat, Cataluña, 08950, Spain	CEIC Hospital Universitari Vall d Hebron	Passeig de la Vall d Hebron 119-129, Institut de Recerca Ed Maternoinfantil Pl 13, Barcelona, Cataluña, 08035, Spain
58003	Hospital Universitari Vall d Hebron	CEIC Hospital Universitari Vall d Hebron	Passeig de la Vall d Hebron 119-129, Institut de Recerca Ed Maternoinfantil Pl 13, Barcelona, Cataluña, 08035, Spain	CEIC Hospital Universitari Vall d Hebron	Passeig de la Vall d Hebron 119-129, Institut de Recerca Ed Maternoinfantil Pl 13, Barcelona, Cataluña, 08035, Spain
60002	Universitaets-Kinderspital	Kantonale Ethikkommission Zuerich	Stampfenbachstrasse 121, Zuerich, 8090, Switzerland		
60002	Universitaets-Kinderspital	Ethikkommission Nordwest und Zentralschweiz	Hebelstrasse 53, Basel, 4056, Switzerland		
66005	Riley Hospital for Children	Indiana University Institutional Review Board	980 Indiana Avenue, Lockefield Room 3338, Indianapolis, IN, 46202, United States		
66009	Cincinnati Children’s Hospital Medical Center	Cincinnati Children’s Hospital Medical Center Institutional Review Board	3333 Burnet Avenue, MLC 5020, Cincinnati, OH, 45229, United States		
66010	Nationwide Children’s Hospital	Nationwide Children’s Hospital Institutional Review Board	700 Children’s Drive, 5th Floor, Columbus, OH, 43205, United States		
