# Supplementary figures and images for: A phase 1, first-in-child, multicenter study to evaluate the safety and efficacy of the oncolytic herpes virus talimogene laherparepvec in pediatric patients with advanced solid tumors
Source: Front Pediatr. 2023 May 24;11:1183295. doi: 10.3389/fped.2023.1183295 (PMC10244735; doi:10.3389/fped.2023.1183295)

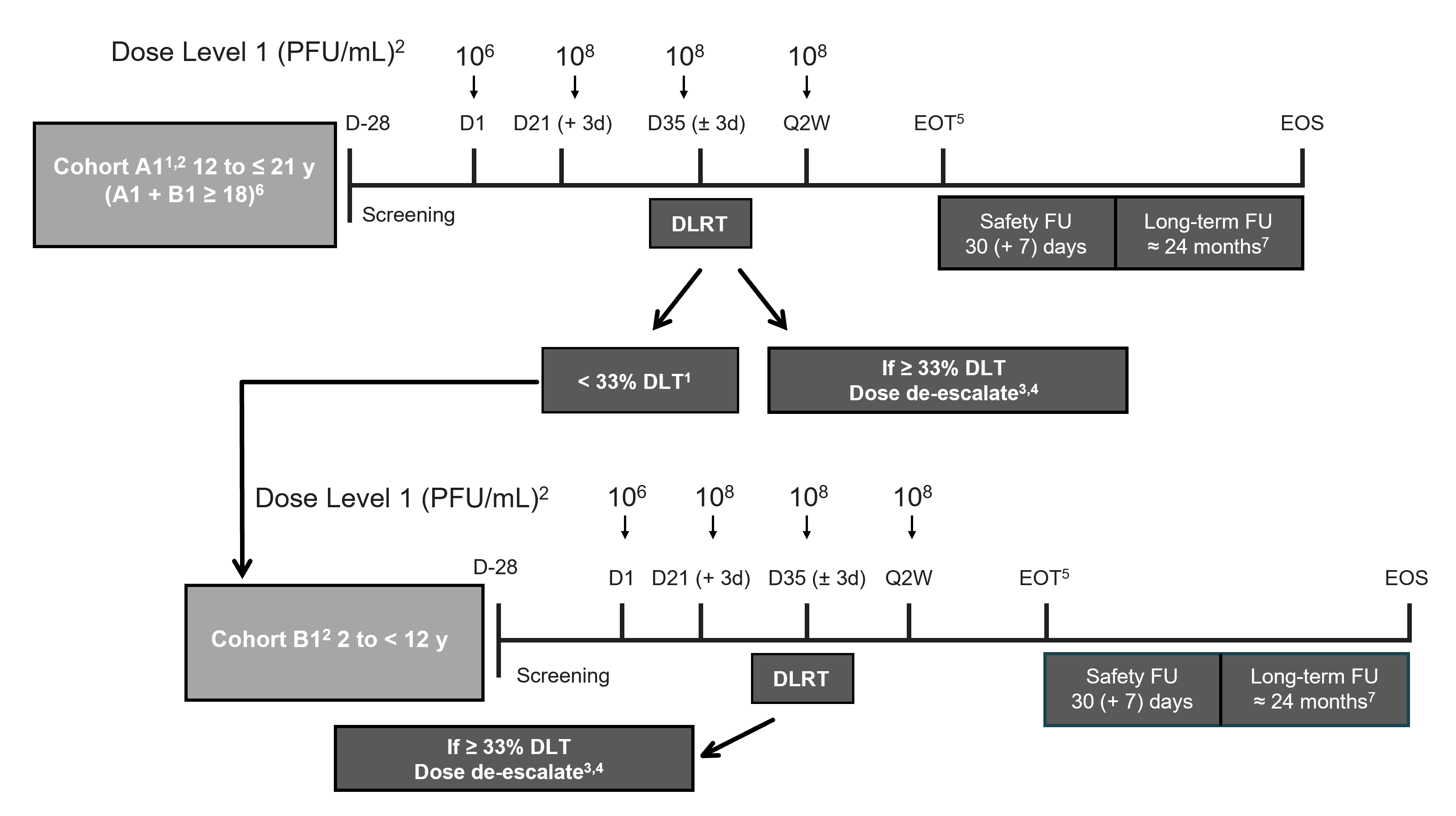

Supplement: Supplementary file 2 [file Image1.tif]

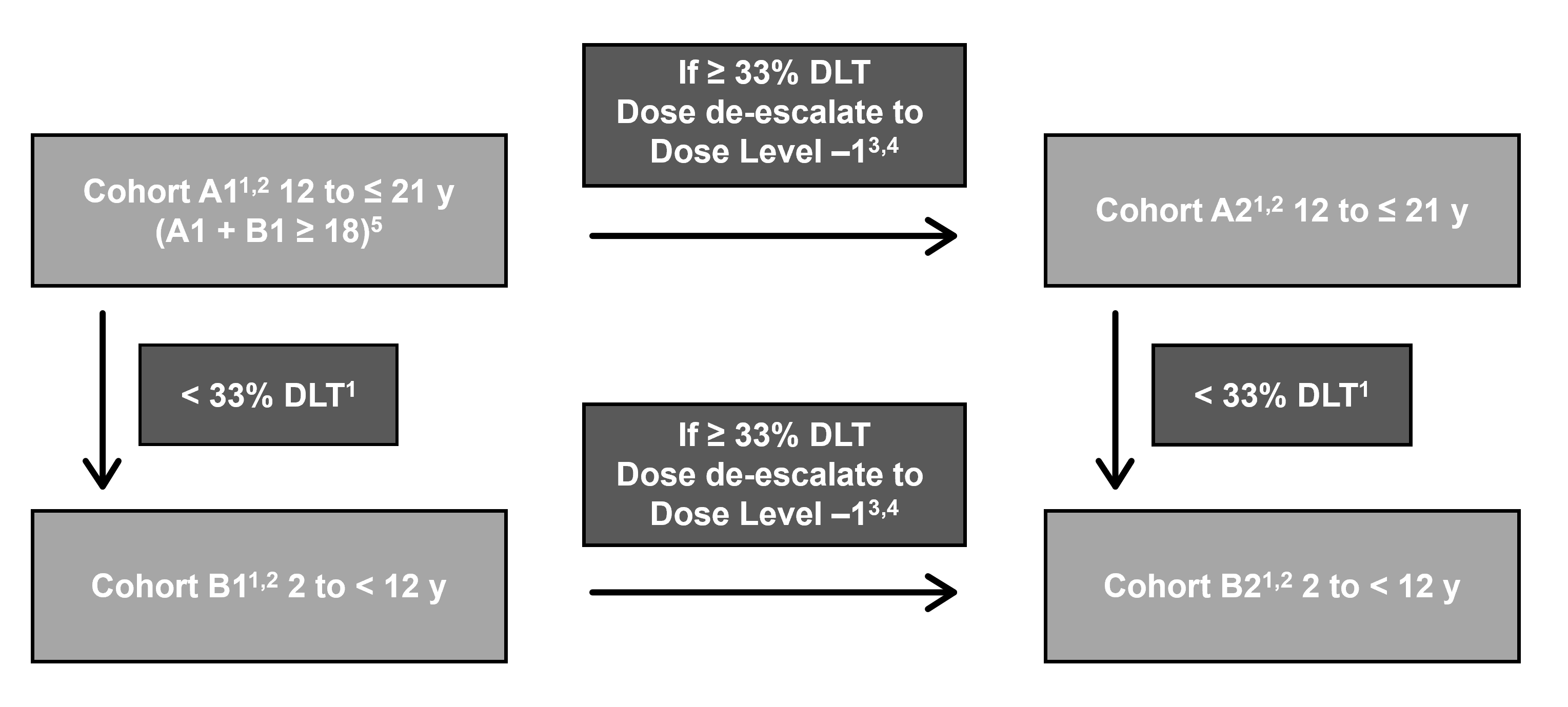

Supplement: Supplementary file 3 [file Image2.tif]

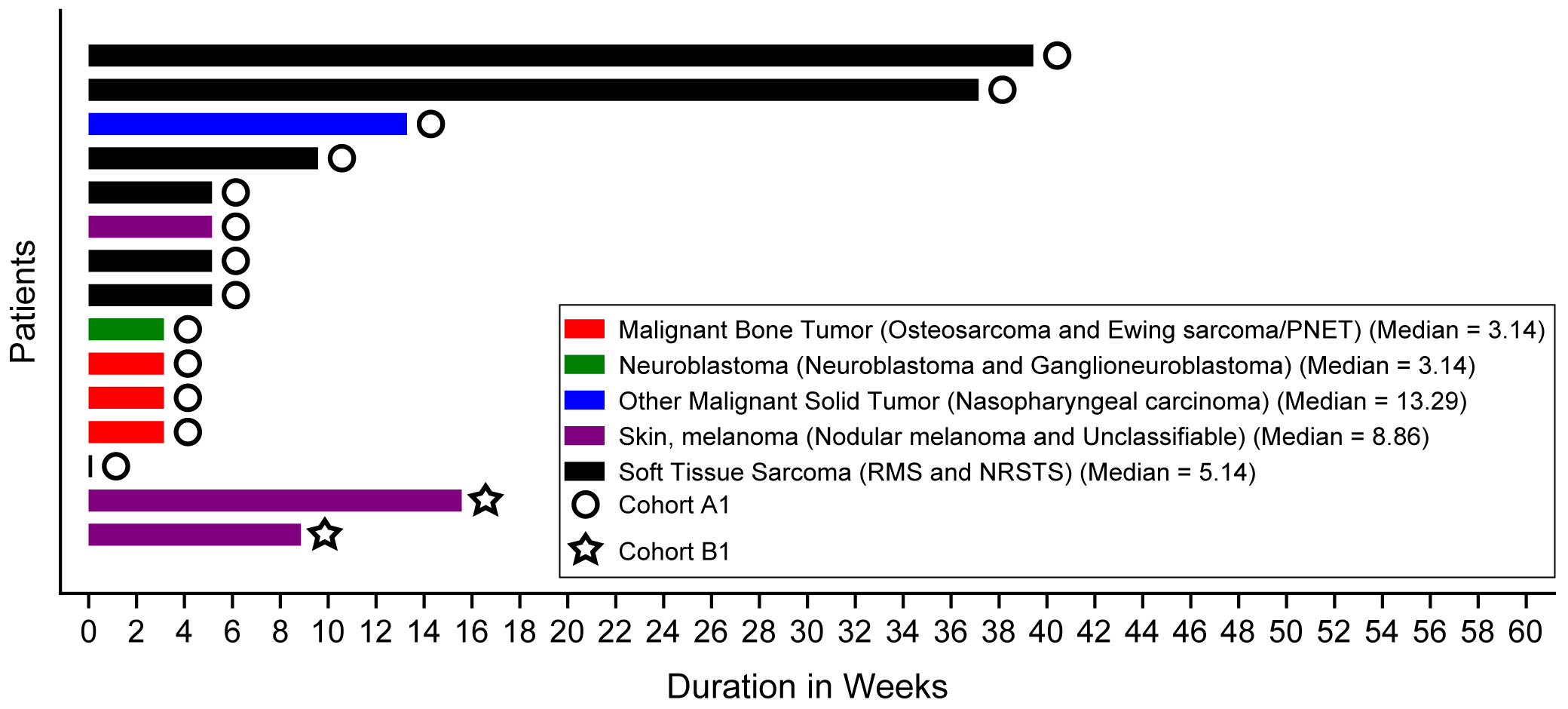

Supplement: Supplementary file 4 [file Image3.tif]

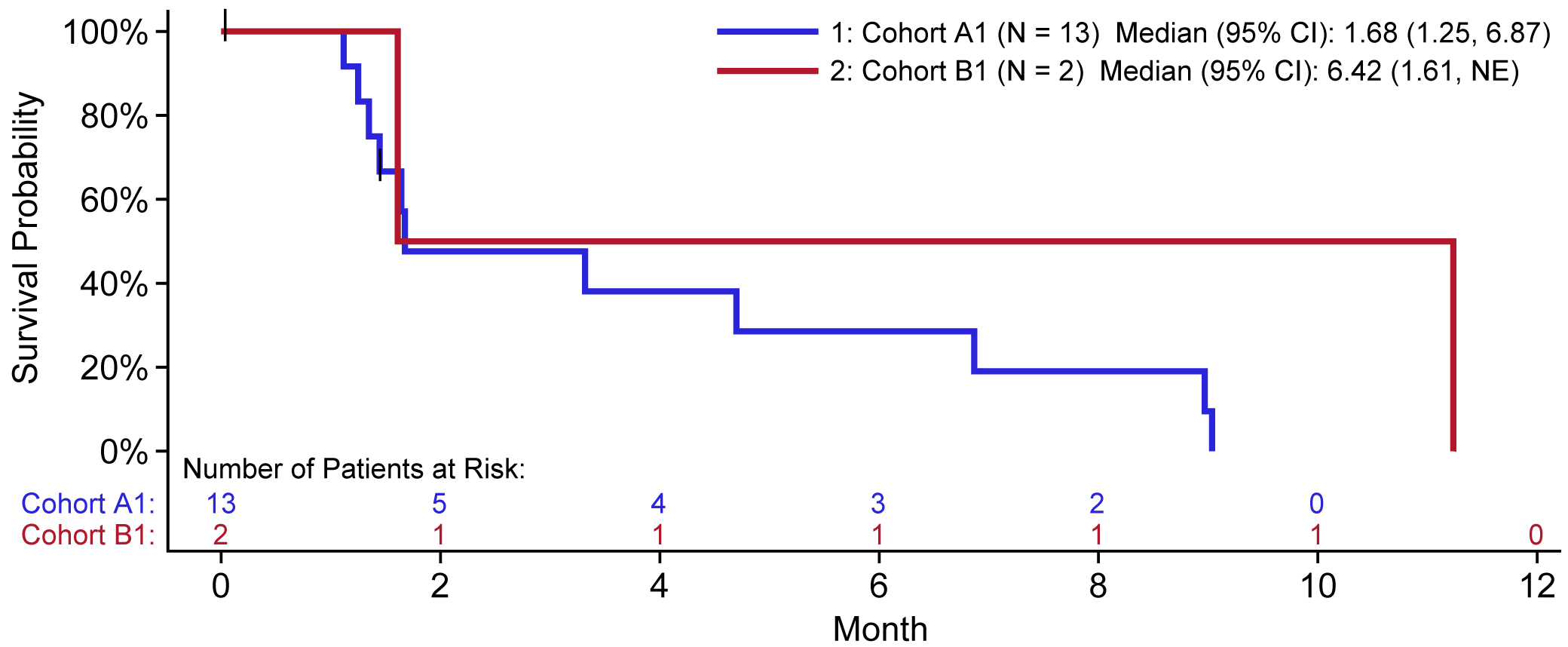

Supplement: Supplementary file 5 [file Image4.tif]
